# Exploring perceived restoration, landscape perception, and place attachment in historical districts: insights from diverse visitors

**DOI:** 10.3389/fpsyg.2023.1156207

**Published:** 2023-08-02

**Authors:** Jiaying Li, Junjie Luo, Tangmin Deng, Jingwen Tian, Hongcheng Wang

**Affiliations:** ^1^Department of Landscape Architecture, Tianjin University, Tianjin, China; ^2^College of Landscape Architecture, Zhejiang A&F University, Hangzhou, China; ^3^The Bureau of Agriculture and Rural of Chengdu Wenjiang District, Chengdu, China

**Keywords:** perceived restoration, place attachment, historical district, structural equation model, landscape perception, visitor groups

## Abstract

Improving the quality of the built environment to enhance people’s mental health is gaining traction across various fields, precipitating valuable actions on the wave of “Healthy China 2030” initiative. While ample studies have confirmed the benefits of interaction with natural or green spaces, the investigation into the restorative potential in urban built settings remains notably underexplored. In this study, we focused on historical districts, conducting a questionnaire survey to evaluate the restorative experiences of individuals visiting these sites. We used Partial Least Square-Structural Equation Modelling (PLS-SEM) to analyze a conceptual model that encompasses landscape perception, place attachment, and perceived restoration, with a specific focus on detecting the mediating role of place attachment and the moderating influence of visitor groups. The results showed that landscape perception significantly influenced the perceived restoration, which contained the indirect effect pathway through place dependence and place identity, as well as the potent direct impact of landscape perception. Moreover, employing a multi-group analysis (MGA), we discerned that different visitor groups partially moderate the relationship between landscape perception, place attachment, and perceived restoration. This study validates the restorative features in historic districts and highlights the importance of cognitive-emotional bond in promoting psychological restoration.

## 1. Introduction

The process of rapid urbanization has resulted in multifaceted challenges to urban planning and designing, particularly in creating livable environments conductive to human health (Haaland and van den Bosch, 2015). These challenges, which include overpopulation, social isolation, traffic-induced air pollution, and the urban heat island effect, have adversely affected the well-belling of urban residents, leading to both physical and mental illnesses (Haaland and van den Bosch, 2015; Feltynowski et al., 2018). Despite a growing body of evidence demonstrates the positive association between urban green spaces and human mental health, the availability of these crucial spaces is progressively diminishing due to high-density urban development (Hartig et al., 2003; Nordh et al., 2009; Grahn and Stigsdotter, 2010). Consequently, the opportunities for engaging in green recreational activities are becoming increasingly limited (Haaland and van den Bosch, 2015; Lin et al., 2015). Therefore, there is a growing awareness of the need to find alternative open spaces and develop infrastructure in urban built environments that prioritize health promotion (Stigsdotter et al., 2017; Subiza-Pérez et al., 2021). This imperative requires immediate attention rather than deferral to the future.

Perceived restoration refers to the progress of improving emotional, physical and attentional states by relieving cognitive fatigue and emotional disorder (Kaplan, 1993; Kaplan, 1995). Although empirical studies suggest a much stronger stress-reductive capacity of natural environments compared to urban settings (Kaplan, 1995; Grahn and Stigsdotter, 2010; Van den Berg et al., 2010), it is important to note that the restorative experience is not solely connected with natural elements (Weber and Trojan, 2018). Indeed, positive emotional support can occur in various types of environments, as long as these settings would meet the requirements of a restorative environment (Kaplan, 1995; Scopelliti et al., 2019). Evidence is emerging that a well-designed urban setting also has a beneficial effect similar to that of nature (Kaplan et al., 1993; Gascon et al., 2017; Xu et al., 2018). For example, the monastery could provide an opportunity to experience mental restoration from a spiritual release perspective (Ouellette et al., 2005). The local history square may offer pleasure and relaxation comparable to that found in urban parks (Fornara and Troffa, 2009). As a place of healing, cultural heritage sites could improve tourist satisfaction and restorative experiences (Cho et al., 2016), with the integration of more water features and flat terrains significantly contributing to the restoration of such cultural landscapes (Xu et al., 2018). Historical districts, combining traditional features, cultural characteristics, and local traits, deeply rooted in the urban memory of the locality (Naoi et al., 2011). As essential components of cultural heritage, these districts not only witness urban development and culture evolution, but also play a vital role in the promotion of urban renewal, enhancement of public environments, and refinement of supporting facilities (Lu et al., 2015; Wang and Aoki, 2019). However, to our knowledge, there is still a lack of empirical research on this type of place to reveal the possibility of the perceived restoration effect.

In addition, a large body of research highlights the materiality of both tangible and intangible factors that may influence restorative experiences (Herzog et al., 2003; Nordh et al., 2009; Grahn and Stigsdotter, 2010; Dallimer et al., 2012). On the one hand, the impact of environmental perception and preference in facilitating individual perceived restoration has been extensively recognized (Herzog et al., 2003; Nordh et al., 2009; Grahn and Stigsdotter, 2010). Dallimer et al. (2012), for example, proposed that the psychological restoration experienced by visitors correlates more with their perceived species diversity rather than the actual species diversity within urban green spaces. Similarly, Subiza-Pérez et al. (2019) indicated that esthetic enjoyment serves as a significant motivation for visiting urban green and blue spaces, with crucial implications for perceived restoration and engagement in physical activities. Masullo et al. (2021a) reported that adding water installations with combined auditory and visual elements can greatly enhance individual restorativeness in environments with background traffic noise. On the other hand, the emotional connections fostered through human-land interactions also play an essential predictive role in restorative environments (Menatti et al., 2019; Subiza-Pérez et al., 2020). The stronger the place attachment to a given place, the more profound the experience of restorative outcomes (Menatti et al., 2019). The psychological enjoyment derived from a place is directly associated with the recovery experience (Korpela et al., 2001). Arguably, perceived restoration is an intricate combination of environmental perception, human cognition, and mental emotion (Scopelliti and Vittoria, 2004).

Moreover, emotional attachment belongs to the dynamic response variable for visitors, exhibiting potential subject disparities (Lewicka, 2010). For example, place attachment tends to be more intensely experienced by visitors who live in close proximity than by those from more distant locations (Kil et al., 2015), and it is also positively related to the length of residence (Brown and Raymond, 2007). Historical districts offer more than just opportunities for tourists to immerse themselves in local history and cultural activities. They also serve as indispensable venues for outdoor recreation for local residents (Wang and Aoki, 2019). Given the potential differences in place attachment between residents and tourists, it is necessary to analyze whether these disparities affect their restorative experiences in historic districts.

With the proposal of “Healthy China 2030” strategic initiative, improving public health has become an essential concern within Chinese society (Tan et al., 2017). Mental health, representing an indispensable dimension of overall well-being, is a breakthrough for ensuring the public have access to healthy life. Considering the above issues, this study aims to systematically understand the supportive qualities that contribute to restorative experiences within the context of historical districts. First, we examined the influence paths between landscape perception, place attachment, and perceived restoration. Second, we detected whether the place attachment could play a mediating role between landscape perception and perceived restoration. Third, we investigated the moderating impact of different visitor groups for the overall conceptual model. We believe the findings could provide an insight into the debate regarding restorative urban built environment, while also providing references for the preservation management and renewal development in historical districts.

## 2. Literature review and conceptual framework

### 2.1. Landscape perception and perceived restoration

According to the Attention Recovery Theory (ART), engaging in many activities that require effortful attention and lead to mental fatigue when individuals’ capacity becomes depleted due to excessive use (Kaplan, 1995). ART identifies four key features of a restorative environment that improve psychological restoration and stress alleviation (Kaplan and Kaplan, 1989; Hartig et al., 1997): ‘being away’ (flighting from daily routine life and exhausted things), ‘fascination’ (engaging attention through specific environmental objects and features), ‘compatibility’ (alignment between personal intentions and environmental activities) and ‘extent’ (immersing oneself into a context distinct from the current one, including both tangible and intangible elements). The environment with ‘extent’ does not necessarily require a large area of space but should be coherent and have a sense of depth. Therefore, the ‘extent’ can be measured by the property of ‘coherence’ and ‘scope’ (Hartig et al., 1997; Pasini et al., 2014; Celikors and Wells, 2022). The ‘coherence’ means an individual’s perception of harmony within the environment, while ‘scope’ refers to the scale of the environment and what can be achieved there (Hartig et al., 2003; Pasini et al., 2014).

Landscape perception refers to the process by which the human brain acquires environmental information through the sensory system, subsequently followed by emotional processing of these information (Purcell, 1987; Mesch and Manor, 1998; Kyle et al., 2004). In other words, landscape perception relies on subjective feelings and psychological evaluations of environment surroundings, also addicted to the psychological basis of people’s environmental behavior (Purcell, 1987; Tschacher et al., 2012). Several studies have explored the indicators and dimensions of landscape perception that can be measured and evaluated across various urban settings (Palacio Buendía et al., 2021). For example, Gobster and Westphal (2004) investigated the human recreation experiences in the urban greenway, utilizing cleanliness, naturalness, esthetics, safety, and access as evaluative indicators. Sotoudeh and Abdullah (2013) summarized six cognitive properties associated with historical buildings, they are coherence, friendly, novelty, complexity, meaningful, and pleasant. In historical districts, visitor evaluations are significantly influenced by key perception features such as touristic, safety, calm, lively, unique, and famous (Naoi et al., 2006). Besides, the unique architectural facades have a positive impact on visitors’ psychological impression. The building styles and their cultural atmosphere can trigger ‘romantic’ emotions and states of mind in visitors (de Freitas et al., 2021).

Regarding the link between landscape perception and perceived restoration, subjective landscape perception has been widely identified as significantly correlated with psychological restoration (Ojala et al., 2019; Malekinezhad et al., 2020). For example, Grahn and Stigsdotter (2010) proposed eight perceived sensory dimensions in urban parks as most pertinent to stress relief, including culture, nature, prospect, refuge, rich in species, social, space and serene. Mahdieh et al. (2011) found that visual perception preferences for urban natural landscapes greatly enhanced their restorative experience. In addition, in urban environments with historical and cultural characteristics, landscape perception is also closely related to the restorativeness (Hidalgo et al., 2006; Fornara and Troffa, 2009). The work of Hidalgo et al. (2006) informed that esthetic preference for history places contributes to the creation of a mental recovery experience. Fornara and Troffa (2009) confirmed that historic sites and urban parks have similar restorative qualities and characteristics. Masullo et al. (2021b) indicated that historical–artistic features present in historical sites are closely related to all the components of restorativeness, particularly the component of fascination. Liu et al. (2019) indicated that a positive soundscape perception in historic blocks could contribute to visitor satisfaction during the visiting experience. In light of these findings, it is inferred that landscape perception could enhance restorative experiences by processing environmental features-related information. Moreover, historical districts, with their unique esthetic characteristics and emotional attributes, also could support perceived restoration for visitors. The following hypothesis thus was proposed:

*H1*: Landscape perception has a significant positive effect on perceived restoration.

### 2.2. Landscape perception and place attachment

Place attachment refers to the connection individuals establish with their environment, encompassing the interaction of emotion, perception, and behavior (Williams and Vaske, 2003). It not only carries conceptual significance in explaining the intrinsic connection between individuals and specific places but also has application value in rebuilding positive emotional bonds (Raymond et al., 2010; Ujang and Zakariya, 2015). The classical two-dimensional structure of place attachment (i.e., place dependency and place identity) has been proven reliable and universal in environmental psychology research (Williams and Vaske, 2003; Jorgensen and Stedman, 2006; Liu et al., 2020). Place dependence emphasizes the physical attachment and functional dependence on facilities or settings, reflecting the specific conditions in which the environment supports the individual’s activities (Jorgensen and Stedman, 2006; Brown and Raymond, 2007). Place identity is part of personal identity, which depends on the typical elements of a specific area and the special sense of belonging (Wester-Herber, 2004; Hernández et al., 2007). Notably, factors such as place-related memories, social interactions, and leisure activities are associated with the formation of place attachment (Ratcliffe and Korpela, 2016; Hosseini et al., 2021). Besides, place attachment also influences behavioral intentions, neighborhood belonging, and psychological recovery (Kyle G. T. et al., 2003).

In addition, studies on cognitive perception and human-land interaction started to increase in past decades (Mesch and Manor, 1998; Bratman et al., 2012; Hosseini et al., 2021). Mesch and Manor (1998) found that a high evaluation of physical and social environment usually represents a greater potential for place attachment. Stedman (2002) argued that place attachment relies on the understanding of the environment and can be fostered only when ‘the feeling’ occurs in the place. Bratman et al. (2012) indicated that the psychological benefits of nature experiences were presumed to be the result of a cognitive process in the specific environment. In other words, place attachment is a part of the self-psychological framework, where cognition and emotion connect individuals to their environment (Fiske et al., 2007). In terms of historical landscape, understanding perception and satisfaction of visitors is a foundation for better protecting and managing cultural heritage sites (de Freitas et al., 2021). Based on the above evidence, this study assumed that the landscape perceptions could induce different levels of human-land emotional responses in historical districts. Hence, we formulated the following hypotheses:

*H2*: Landscape perception has a significant positive effect on place attachment.

*H2a*: Landscape perception has a significant positive effect on place dependence.

*H2b*: Landscape perception has a significant positive effect on place identity.

### 2.3. Place attachment and perceived restoration

The relationship between place attachment and perceived restoration has been confirmed in recent studies (Menatti et al., 2019; Kastenholz et al., 2020). People find it easier to spend time and obtain relaxing experiences in places with high attachment levels (Kyle G. et al., 2003; Lewicka, 2008; Ratcliffe and Korpela, 2016). Thus, fostering stronger place attachment bonds among visitors could positively impact psychological states (Pretty et al., 2016). Korpela et al. (2001) reported that the enjoyment of a place is directly proportional to the recovery experience. Ratcliffe and Korpela (2016) found that the memory properties displayed a predictive effect on perceived restoration, with place attachment as a mediating variable. Additionally, regarding the sub-dimensions of place attachment, both place identity and place dependence were consistently associated with perceived restoration (Ratcliffe and Korpela, 2016). Liu et al. (2021) suggested that place identity is a more potent predictor of restorativeness in urban parks compared to place dependence. These outcomes make it evident that restorative experiences are related to the individual’s affective attitudes; thus, the following hypotheses were tested:

*H3*: Place attachment has a significant positive effect on perceived restoration.

*H3a*: Place dependence has a significant positive effect on perceived restoration.

*H3b*: Place identity has a significant positive effect on perceived restoration.

### 2.4. Place dependence and place identity

It is also important that place dependence and place identity were initially often studied side by side. However, as research progressed, scholars became aware of the potential recursive relations between identity and dependence (Stedman, 2002; Hernández et al., 2007; Liu et al., 2020). Halpenny (2010) found that the impact of place dependence on visitors’ pro-environmental intentions is mediated by place identity. Similarly, Wan et al. (2022) proposed that place dependence indirectly influences recycling intention through place identity. That is to say, people are more likely to develop a sense of emotional identification and belonging (i.e., place identity) if one place can provide the physical conditions and characteristics to meet individual needs (i.e., place dependency) (Trąbka, 2019; Wan et al., 2022). Based on the above outcomes, there is also a possibility of precedence from place dependence to place identity in visiting historical districts. Thus, we proposed following hypothesis:

*H4*: Place dependence has a significant positive effect on place identity.

### 2.5. The moderating effect of visitor groups

Regarding the participants, status on environment perception and emotional connection, several studies have confirmed the different perspectives between residents and visitors (Home and Vieli, 2020; Dasgupta et al., 2022; Guo et al., 2022). For example, Vaz de Freitas et al. (2021) discovered that residents value heritage conservation and transport mobility more in urban landscape, while visitors place more importance on pedestrian mobility and esthetic quality. Giannakopoulou et al. (2011) employed contingent valuation surveys to assess the perceptions and attitudes of local residents and visitors toward the preservation of traditional architecture. The findings revealed that residents exhibited greater awareness of the actual extent of architectural decay and demonstrated a higher level of concern for its protection. As historical and cultural districts play a crucial role in leisure living spaces for residents and visiting places for tourists, the status of different visitors may have a moderating role in the influencing mechanisms of landscape perception, place attachment, and perceived restoration. Thus, we proposed the following hypotheses:

*H5*: Visitor groups have a significant moderating effect on the relationships between landscape perception and perceived restoration (H5a); landscape perception and place dependence (H5b); landscape perception and place identity (H5c); place dependence and perceived restoration (H5d); place identity and perceived restoration (H5e); place dependence and place identity (H5f).

**Figure 1:**
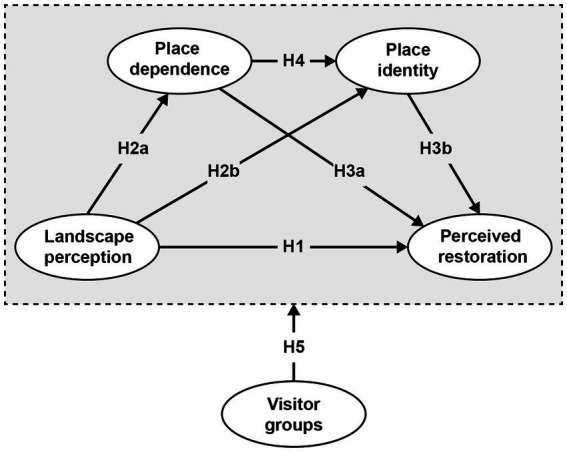
Conceptual framework.

In summary, the aim of this study is to systematically investigate the restorative qualities of historical districts that influence the perceived restoration experienced by diverse visitor groups. The research developed a conceptual model to analyze the connections between landscape perception, place attachment, and perceived restoration; meanwhile, a PLS-SEM was employed to detect these relationships (Figure 1).

## 3. Methodology

### 3.1. Study area

**Figure 2:**
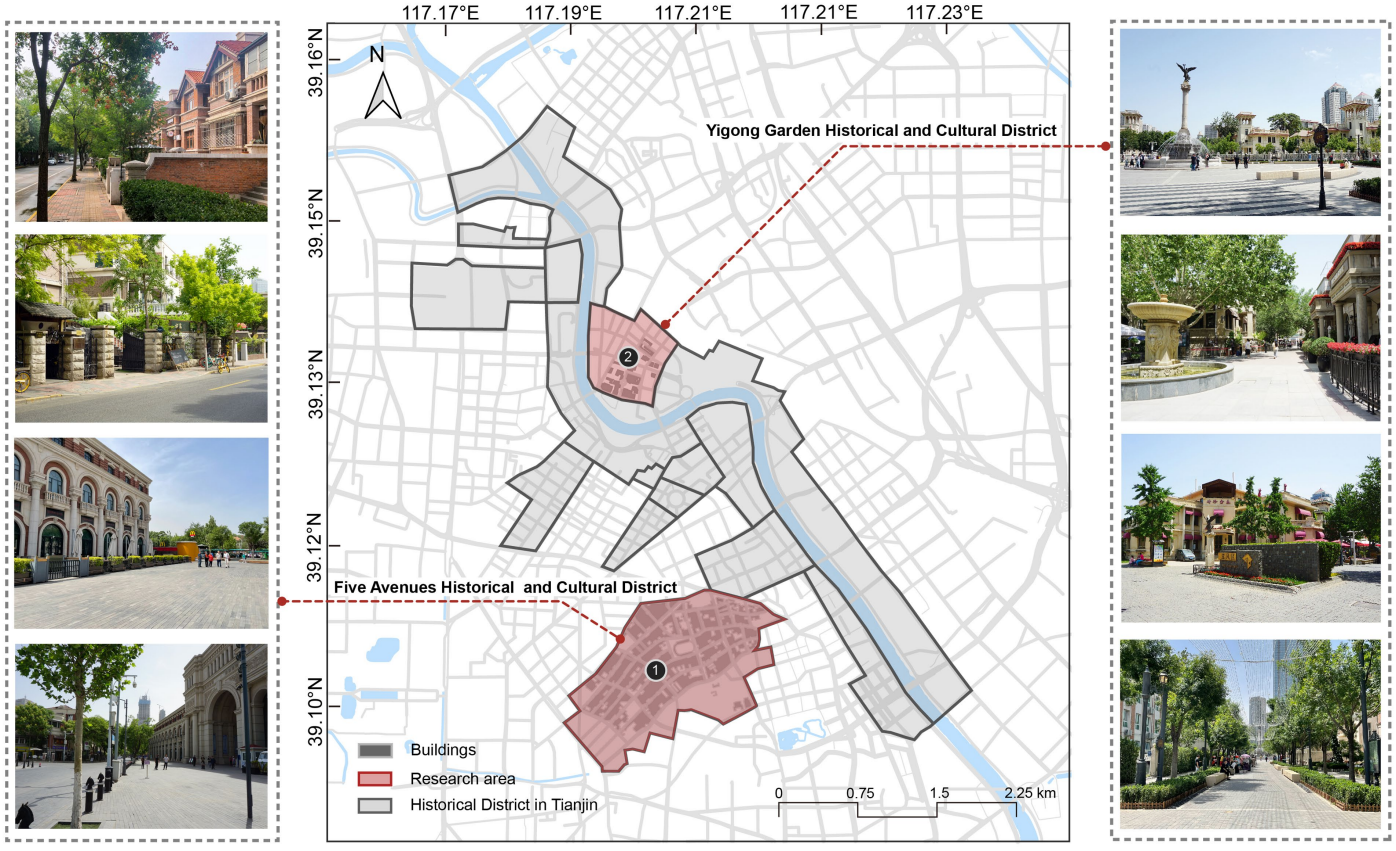
Study area and field photographs of the two selected historical districts (Base map: Reproduced with permission from https://map.baidu.com. City photos: Taken by the authors).

Tianjin, located in northern China, is an early city with historic connections to Western civilization. Since it was established as an important commercial port in 1860, the city has built and reserved many exotic-style buildings along Haihe river. Over time, these buildings and their surroundings gradually merged with the city’s development plans, culminating in unique historical and cultural districts. These districts now stand as prominent landmarks in Tianjin, representing the city’s distinctive heritage and symbol. In this study, two typical historical districts were selected as sample sites. One is the Five Avenues Historical and Cultural District, and another is the Yigong Garden Historical and Cultural District (Figure 2). The selection criteria for these sites were as follows. First, the two sites serve as dual roles for tourism and leisure, meeting the requirements of both daily recreation and cultural experiences. Second, these locations maintain distinctive architectural features and well-preserved streetscapes, drawing a large number of visitors for sightseeing and touring.

### 3.2. Questionnaire design

Based on previous studies (Pazhouhanfar and M.s. MK., 2014; Home and Vieli, 2020; Liu et al., 2020; Malekinezhad et al., 2020), we formulated a questionnaire, including four sections and 30 statements. The questionnaire was translated from English into Chinese and subsequently pre-tested by employing twenty college students to ensure its acceptability and accuracy. The four parts are as follows:

*Demographic characteristics*: The factors in this part contained gender (male and female), age (<18, 18–40, 41–65, >66), education (below high school, high school, undergraduate, and above undergraduate), occupation (student, employed, unemployed, and retired), and visitor status (long-time residents, short-time residents, and transient tourists). Long-term residents in Tianjin are defined as individuals who have lived in the city for a minimum period of six months and fulfill one of the following requirements: legal employment, stable residence, or continuous education. Short-time residents encompass individuals who have temporarily settled in Tianjin for at least one month and are engaged in work or business activities. Lastly, transient tourists pertain to individuals who visit Tianjin for a short period solely for tourism purposes.

*Perceived Restorativeness Scale*: Considering the integrity and efficiency of the evaluation, this study used a concise version of the Perceived Restorativeness Scale (PRS-11) as proposed by Pasini et al. (2014). The PRS-11 scale is condensed to collect data in a shorter time and regardless of gender or nationality, which has been widely applied in the evaluation of various restorative environments, such as urban garden (Home and Vieli, 2020), cemetery (Nordh et al., 2017), and campus (Shrestha et al., 2021). This scale includes eleven statements in four dimensions: ‘Fascination’, ‘Coherence’, ‘Scope’, and ‘Being away’. Likert seven-point scales were used for this scale.

*Place Attachment Scale*: Given that the classical two-dimensional structure of place dependence and place identity is widely applied, we employed an eight-item scale across two dimensions, following their successful utilization in previous studies (Williams and Vaske, 2003; Lewicka, 2008; Hosseini et al., 2021). Specifically, four items were designed to measure place dependence and four items to measure place identity, with some minor adjustments to better fit the context of the historical district (Williams and Vaske, 2003; Kyle et al., 2005). Likert seven-point scales were used for this scale.

*Landscape Perception Evaluation*: This section addressed the subjective evaluation of landscape perception in historical districts, using widely validated indicators from previous studies (Gobster and Westphal, 2004; Grahn and Stigsdotter, 2010; Tempesta, 2010; Liu et al., 2019). We focused on six dimensions of perception evaluation, which include ‘esthetic’, ‘historical’, ‘unique’ ‘pleasant’, ‘natural’, and ‘safety’. Each item was measured with seven-point semantic differential scales using the evaluative adjective.

### 3.3. Data collection and analysis

Data were collected from November 2020 to May 2021 on days with good weather conditions. The participant was randomly selected from visitors who had actually visited the historical districts to ensure the collected data accurately represented authentic visiting experiences. All participants were informed by the interviewers about the purpose of the study, the research methodology, the affiliations of the institution, and why their participation was essential. They were further assured of the anonymity of their responses and the confidentiality of the information they provided. Before participating in the study and filling out the questionnaire, participants confirmed they understood the instructions and consented to their involvement in the research.

A total of 620 questionnaires were given out, and 564 responses were returned, representing a 90.96% response rate. The data was processed in SmartPLS 4.0, and we organized follow steps for the statistical analysis. Firstly, we tested the validity and reliability of each item. Secondly, we used PLS-SEM to detect the relationships between landscape perception, place attachment, and perceived restoration. PLS-SEM is a comprehensive approach that can examine complex models with direct and indirect relationships, which is suitable for the research of main concerns are theory development and prediction (Hair et al., 2019). Thirdly, we analyzed the mediating role of place attachment in the generation mechanism of perceived restoration. Finally, the Multiple Group Analysis (MGA) was applied to examine the difference between the three visitor groups.

## 4. Results

### 4.1. Descriptive statistics characteristics

**Figure 3:**
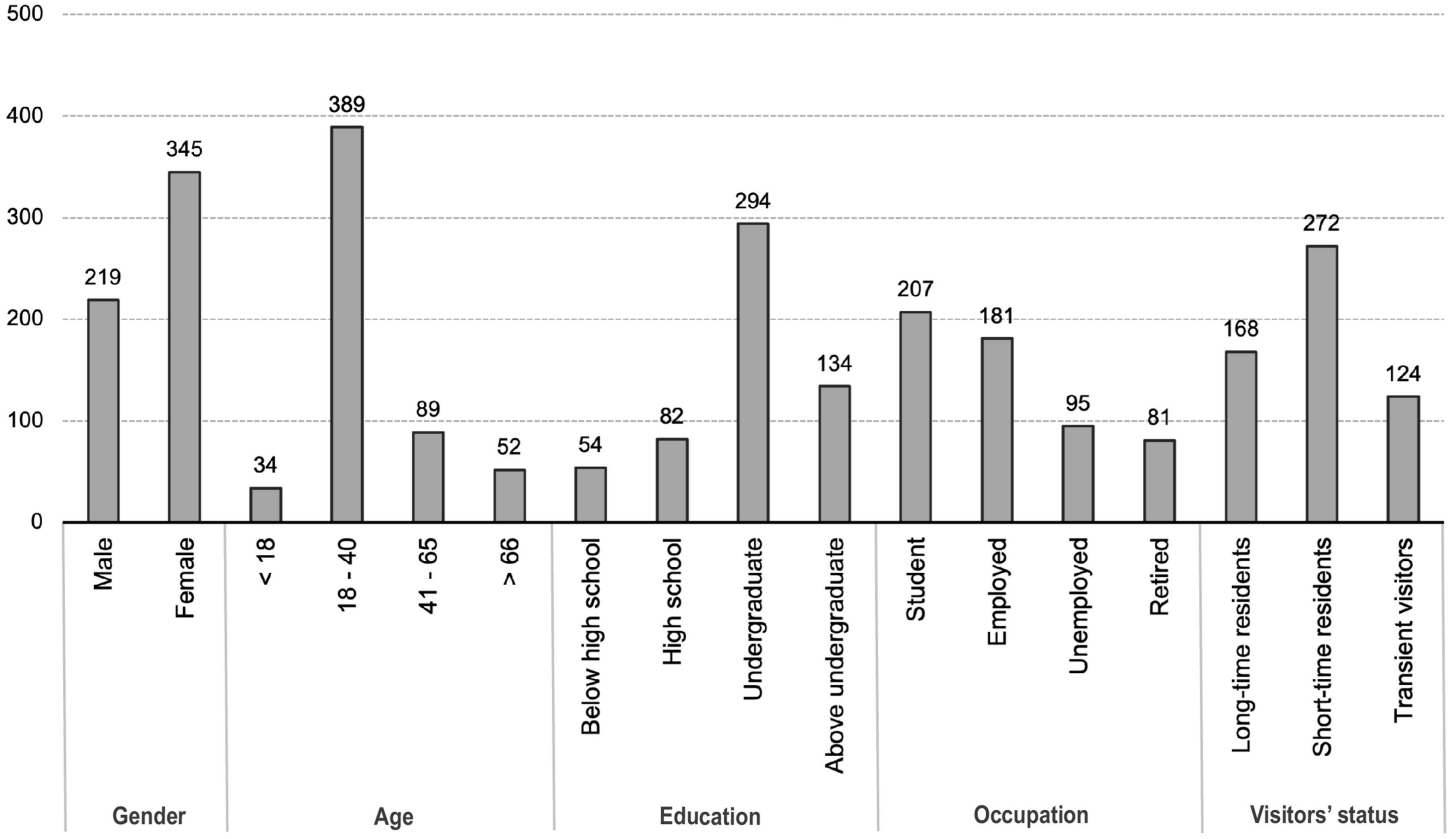
Demographic characteristics of the participants in this questionnaire.

The demographic characteristics of the participants in this questionnaire are shown in Figure 3. The participant consisted of 61.2% females and 38.8% males. Regarding age, 69.0% of participants in the sample were 18 to 40 years old, 6.1% were below 18 years old, and 24.9% were aged 41 years old and above. Regarding education attainment, participants with undergraduate degrees (52.1%) and above undergraduate education backgrounds (23.7%) occupied a large proportion. For occupation differences, participants of the student, employed, unemployed and retired accounted for 36.7, 32.0, 16.8, and 14.5%. In terms of visitors’ status, the majority of participants were short-time residents (48.2%), followed by long-time residents (29.8%), and transient tourists (22.0%).

### 4.2. Reliability and validity

Table 1 presents the reliability and validity for each item. Firstly, Cronbach’s alpha coefficient (α) was applied to test the reliability of the questionnaire. The results indicated good internal reliability and stability, with the α values ranged from 0.813 to 0.892, all exceeding the recommended threshold of 0.8 (Shevlin et al., 2000). Secondly, the validity of the questionnaire was examined using average variance extracted (AVE) and combined reliability (CR), with 0.5 and 0.7 as the reference threshold, respectively (Hair et al., 2010). The analysis showed that AVE values ranged from 0.589 to 0.842, while CR ranged from 0.889 to 0.931. These results indicate good convergent validity and support the interpretation of each item, making the questionnaire suitable for further application in structural equation modeling.

**Table 1:** Construct reliability and validity analysis.

Variable	Item	Standard deviation	Cronbach’s α	AVE	CR
	Place attachment				
Place dependence	Pd1 When I want to do outdoor recreation, this is the best place I go to.	0.848	0.845	0.683	0.895
	Pd2 This place is more worth visiting than other places.	0.701			
	Pd3 The leisure activity experience here is more satisfying than anywhere else I have been.	0.884			
	Pd4 No other place can compare to this place.	0.860			
Place identity	Pi1 I have a lot of personal memories link in here.	0.886	0.892	0.756	0.925
	Pi2 I feel this place is an integral part of my life.	0.880			
	Pi3 I identity strongly with visiting in here.	0.843			
	Pi4 I am very attached to this place.	0.868			
	Perceived restoration				
Being away	B1 This district like a refuge where I can feel unconstrained.	0.903	0.889	0.819	0.931
	B2 Spending time in this district gives me a good break from my day-to-day routine.	0.905			
	B3 This district let me get away from things that usually drain my attention.	0.907			
Scope	S1 In this district there are few boundaries to limit my possibility for moving about.	0.916	0.813	0.842	0.914
	S2 This district is large enough, with no restrictions to move movements.	0.919			
Fascination	F1 This district is fascinating and charming.	0.851	0.813	0.728	0.889
	F2 In this district like this my attention is drawn to many interesting things.	0.872			
	F3 The landscape in this district awakens my curiosity.	0.836			
Coherence	C1 In this district is easy to move around so that I could any activities I want.	0.844	0.827	0.744	0.897
	C2 This district like everything seems to have its proper place.	0.895			
	C3 The landscape in this district is organized and arranged.	0.847			
	Landscape perception				
Landscape perception	Lp1 Esthetic (beautiful-unattractive).	0.813	0.860	0.589	0.895
	Lp2 Historical (traditional-modern).	0.754			
	Lp3 Unique (ordinary-distinctive).	0.675			
	Lp4 Pleasant (pleasant-unpleasant).	0.784			
	Lp5 Natural (natural-artificial).	0.774			
	Lp6 Safety (safety-dangerous).	0.796			

In addition, it is worth noting that all indicators exhibited loadings exceeding 0.70, except for Lp3, which had a slightly lower loading of 0.675. However, we retained this indicator since the consideration that weaker outer loadings between 0.40 and 0.70 can be retained if they can explain 50% of AVE (Hair et al., 2019). Besides, to assess discriminant validity, the Fornell-Larcker criterion was employed. This criterion compares the square root of AVE for each construct with the correlations between pairs of latent variables. In this study, all correlation coefficients were found to be smaller than the square root of AVE, indicating an acceptable level of discriminant validity (Table 2).

**Table 2:** Discriminant validity analysis.

	Landscape perception	Place dependence	Place identity	Coherence	Fascination	Scope	Being away
Landscape perception	**0.767**						
Place dependence	0.457	**0.826**					
Place identity	0.381	0.749	**0.870**				
Coherence	0.555	0.458	0.485	**0.862**			
Fascination	0.552	0.475	0.502	0.734	**0.853**		
Scope	0.575	0.460	0.501	0.733	0.745	**0.918**	
Being away	0.593	0.536	0.534	0.745	0.692	0.745	**0.905**

Bold values depict square roots of AVE for each latent variable.

### 4.3. Measurement of the structural model

To analyze the path correlations and statistical significance among variables, we used the bootstrapping resampling and blindfolding approach. Specifically, the coefficient of determination values (R^2^) was calculated to assess the extent to which the variance of a latent variable is explained by its overall variance (Hair et al., 2019). Acceptable R^2^ values for this study were observed for place dependence (0.208), place identify (0.563), and perceived restoration (0.531). To evaluate the model’s predictive relevance for each construct, we considered the predictive relevance (Q2), which indicates the effectiveness of each prediction variable. The Q2 values obtained were 0.139 for place dependence, 0.421 for place identity, and 0.326 for perceived restoration. Additionally, the standardized root mean square residual (SRMR) was utilized to assess the suitability of the measurement model’s evaluation. In this study, the SRMR value was 0.073, which is below the threshold of 0.08 (Hair et al., 2019). This indicates a good fit between the empirical covariance matrix and the theoretical covariance matrix implied by the model.

### 4.4. Hypothesis testing

#### 4.4.1. Analysis of path relationships

**Figure 4:**
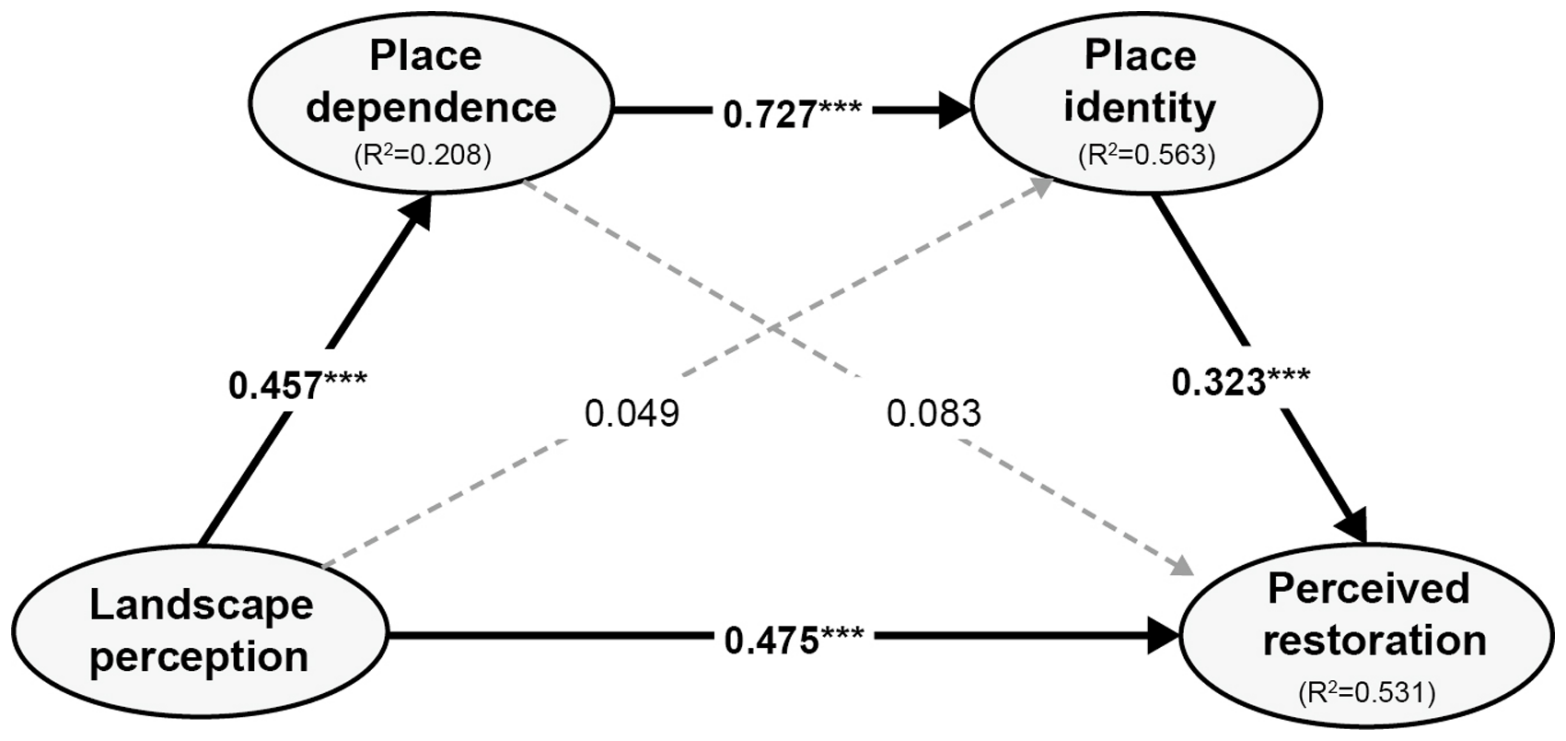
Coefficient values of the conceptual model.

As shown in Figure 4 and Table 3, standardized path coefficients and their significance indicate the relationships among landscape perception, place attachment, and perceived restoration. Specifically. H1 was supported (*β* = 0.475; *p* < 0.001), meaning landscape perception has a positive impact on perceived restoration. Regarding H2 (H2a and H2b), which aimed to examine the impact of landscape perception on place attachment. It was found that landscape perception showed a positive effect on place dependence (*β* = 0.457; *p* < 0.001); thus, H2a was accepted. However, there is no significant association between landscape perception with place identity (*β* = 0.049; *p* > 0.001), with H2b was refused. H3 was designed to detect the path relationship between place attachment and perceived restoration. The results indicated that H3a (the influence of place dependence on perceived restoration) was refused (*β* = 0.083; *p* > 0.001), while H3b (the influence of place identity on perceived restoration) was accepted (*β* = 0.323; *p* < 0.001). H4 was supported (*β* = 0.727; *p* < 0.001), which meant place dependence has a direct significant positive effect on place identity.

**Table 3:** SEM path estimation and significance test.

Hypothesis	Path	Estimate	*T*-value	*p* values	Conclusion
H1	Lp → Pr	0.475***	12.425	0.000	Accepted
H2a	Lp → Pd	0.457***	11.835	0.000	Accepted
H2b	Lp → Pi	0.049	1.478	0.070	Rejected
H3a	Pd → Pr	0.083	1.544	0.061	Rejected
H3b	Pi → Pr	0.323***	6.683	0.000	Accepted
H4	Pd → Pi	0.727***	27.510	0.000	Accepted

Lp, Landscape perception; Pi, Place identity; Pd, Place dependence; Pr, Perceived restoration. **Means *p*-value is significant at 0.05 level, ***Means *p*-value is significant at 0.001 level.

#### 4.4.2. Analysis of mediating effects

The bootstrapping method was applied to test direct and indirect effects with multiple repetition sampling conditions of 2000 samples. Table 4 shows the results of the direct effects, indirect effects, and total effects. Among these, landscape perception had a direct effect on perceived restoration (*β* = 0.475, *p* < 0.001); meanwhile, place attachment showed an indirect effect between landscape perception and perceived restoration (*β* = 0.161, *p* < 0.001). Therefore, the mediation path was as follows: ‘landscape perception → place dependence → place identity → perceived restoration’. However, the effects of the other two mediation pathways (landscape perception → place dependence → perceived restoration; landscape perception → place identity → perceived restoration) were not statistically significant. That is to say, landscape perception could influence the restorative experience in a direct way. Simultaneously, it can induce feelings of place attachment, which in turn facilitate emotional regulation mechanisms that help escape from mental exhaustion ultimately.

**Table 4:** Direct and indirect effects of landscape perception on perceived restoration.

Effect	Path	Estimate	*T*-value	*p* values	Conclusion
Direct	Lp → Pr	0.475***	12.425	0.000	—
Indirect	Lp → Pd → Pr	0.038	1.512	0.065	No mediated
	Lp → Pi→Pr	0.016	1.429	0.077	No mediated
	Lp → Pd → Pi→Pr	0.107***	5.697	0.000	Partly mediated
Total indirect	Lp → Pr	0.161***	6.234	0.000	—
Total effect	Lp → Pr	0.637***	20.068	0.000	—

Lp, Landscape perception; Pi, Place identity; Pd, Place dependence; Pr, Perceived restoration. **Means *p*-value is significant at 0.05 level; ***Means *p*-value is significant at 0.001 level.

#### 4.4.3. Multiple group analysis

H5 predicted that visitor groups moderate the relationships between landscape perception, place attachment and perceived restoration. Before testing the moderating effect, we used MICOM analysis to detect measurement invariance within the structural model across three distinct visitor groups (Henseler et al., 2015). In this case, we adopted a comparative two-group approach, resulting in three comparisons: long-time residents and short-time residents; long-time residents and transient tourists; short-time residents and transient tourists. According to Henseler et al. (2015), the configural invariance was firstly established, which automatically meted the MICOM procedure in SmartPLS 4.0. Second, we calculated compositional invariance, utilizing the original composite correlation value and the 95% confidence interval as a baseline. The results provided substantial support for compositional invariance (Supplementary Table A1). We then applied a 5,000 permutations test to determine whether the composites’ mean values and variances were equivalent (Supplementary Tables A2, A3). In three groups, some permutation variances ratios were outside the 95% confidence interval for the value of the initial differences, leading to the establishment of partial measurement variance.

Multigroup analysis was then performed by comparing the paths across different groups. Path coefficients and significance across visitor groups are presented in Table 5. H5b was supported in terms of the path of ‘landscape perception → place dependence’, which exhibited a more pronounced effect on long-term residents compared to transient tourists. Besides, landscape perception exerted a weak direct effect on place identity for long-time residents, whereas this effect was non-significant for short-time residents and transient tourists. Thus, the hypothesis H5c was supported. Moreover, the path of ‘place dependence → perceived restoration’, varied in different groups, exhibiting significance only in the case of transient tourists, thereby supporting H5d. Concerning the relationship of ‘place dependence → place identity’, long-time residents displayed significantly lower values compared to the other two groups, and hypothesis H5f was supported. Additionally, H5a and H5e were rejected.

**Table 5:** Path coefficient comparison between three groups.

	LR	SR	TT	(LR – SR)		(LR – TT)		(SR – TT)	
	Path coefficient	Path coefficient	Path coefficient	DI	*p* value	DI	*p* value	DI	*p* value
H5a: Lp-Rp	0.483***	0.487***	0.461***	−0.004	0.485	0.021	0.416	0.025	0.378
H5b: Lp-Pd	0.563***	0.449***	0.330***	0.114	0.078	0.233	**0.017**	0.120	0.138
H5c: Lp-Pi	0.164**	0.015	0.070	0.150	**0.038**	0.095	0.184	−0.055	0.266
H5d: Pd-Rp	0.083	0.044	0.212**	0.039	0.375	−0.129	0.189	−0.167	0.113
H5e: Pi-Rp	0.321***	0.291***	0.344***	0.030	0.393	−0.023	0.419	−0.053	0.324
H5f: Pd-Pi	0.589***	0.769***	0.751***	−0.180	**0.003**	−0.162	**0.024**	0.018	0.398

Lp, Landscape perception; Pi, Place identity; Pd, Place dependence; Pr, Perceived restoration; LR, long-time residents; SR, short-time residents; TT, transient tourists. **Means *p*-value is significant at 0.05 level, ***Means *p*-value is significant at 0.001 level. Bold value means there was a significant difference between the two groups, with p-value is significant at 0.05 level.

## 5. Discussion and conclusion

### 5.1. Main conclusion

In our study, we uncovered the association between landscape perception, place attachment and perceived restoration by PLS-SEM, as well as further confirmed the mediating effects of place attachment. By MGA analysis, we analyzed the moderating role of different visitor groups among long-time residents, short-time residents, and transient tourists. Main conclusion from these analyses is described and presented in the following.

Firstly, the landscape perception of visitors in historic districts significantly influenced place dependence. This finding suggests that individuals always rely on their perception of environment to subsequently develop emotional associations. This further confirms the importance of landscape perception as a precursor in developing place attachment. As mentioned by Stedman (2002), place dependence not only sustains emotional ties but also relies on the perception of the psychical environment. In addition, landscape perception did not establish a direct and significant correlation with place identity. This could potentially be attributed to the fact that the formation of place identity requires solid knowledge, familiar understanding, and profound impression, all of which contribute to a sense of belonging (Gu and Ryan, 2008).

Secondly, in this study, as respondents’ scores on place identity increased, they were more inclined to perceive restoration. This finding is consistent with the investigation from Liu et al. (2020), who claimed that place identity had a stronger influence on perceived restorativeness than place dependence. Similarly, Kyle et al. (2004) indicated that respondents with a high place identity dimension were more likely to support payment for preserving and restoring the natural environment. These findings further supported the critical role of place identity in the process of place attachment influencing restorative perception.

Thirdly, we found that place dependence and identity as sub-dimensions of place attachment were not entirely uniform. Place dependence significantly influenced place identity in historic districts, demonstrating that the formation of place attachment follows a progressive path from functional to emotional attachment. Although interrelated, the two dimensions of place attachment represent different elements of the human-land connection. Indeed, when both place dependency and place identity are mentioned, identity tends to develop subsequent to dependence, revealing a more time-dependent feature (Hernández et al., 2007).

Fourthly, the response path of perceived restoration has a direct effect of ‘landscape perception → perceived restoration’ and a partially mediated effect of ‘landscape perception → place dependence → place identity → perceived restoration’. The pathway suggested that landscape perception can directly trigger restorative experience, as well as indirectly contribute to functional dependence and emotional identification, further facilitating relaxation from worries and stresses. Our findings align with several studies that validate the association between place attachment and entertainment (Adevi and Grahn, 2011; Ratcliffe and Korpela, 2016). Arguably, the bonds between individuals and their environment are inherently tied to the perceived restoration.

Finally, our study reveals the moderating effects of visitor groups on the relationship between landscape perception, place attachment, and perceived restoration. As investigated in previous studies, the relationship between landscape perception and restorative perception was influenced by significant differences in place attachment among tourists of different identities (Gu and Ryan, 2008; Trąbka, 2019). In current study, the landscape perception of long-time residents could directly influence place identity. Transient tourists, in contrast, exhibit a significant direct association between place dependence and perceived restoration, indicating that functional dependence is more important for them. These findings supported the concept that place attachment involves a psychological investment in a locale that tends to evolve over time (Halpenny, 2010; Wan et al., 2022). Place dependence is more pertinent for short-time sightseeing, while place identity is essential for the long-time visitor.

### 5.2. Contribution and implication

#### 5.2.1. Theoretical implications

The theoretical implications of this study are as follows. Firstly, this study established a conceptual model of the correlation between visitors’ landscape perception, place attachment and perceived restoration. With urban densification potentially diminishing the access to green recreational spaces, our results validated the restoration potential of historic districts and extended the application of ART in the urban environment. Secondly, our findings confirmed that perceived restoration is a bottom-up process, achieved by utilizing landscape features and manipulating place attachments to ultimately achieve the experience of restoration. More importantly, these results could improve awareness regarding the integration of the concept of human-land bonds into the design process, aiming to create highly restorative settings in urban areas. These findings are also in line with the recommendations of UNESCO on the management of Historical Urban Landscapes (Ginzarly et al., 2019). Thirdly, we examined the significant moderating effect of the visitor category in conceptual model. Consistent with Jutla (2000), tourists’ perceptions are influenced by the natural and cultural landscapes, whereas residents’ perceptions are shaped by their familiarity with the place. Therefore, concerning and understanding the variations in characteristics among different visitor groups provides an innovative perspective in clarifying the process of mental recovery. This information also appeals more targeted strategies for designing and planning restorative environments.

#### 5.2.2. Management implications

Historical districts enjoy an emerging popularity in China due to their traditional buildings, unique historical accumulation, and diverse cultural connotations. The characteristic of landscape perception identified in our study may provide some practical contributions for urban planners and managers aiming to create appealing presentations of historic districts. Such measures may involve conserving local cultural customs, historic architecture, and symbolic landmarks to present an authentic portrayal. Considerations could also include enhancing the asthetics of street facade, enabling pedestrian safety, and displaying local characteristics to positively aggrandize the landscape evaluation.

In addition, stronger emotional connections toward a place tend to cultivate more positive restorative experiences. Thus, policy managers should formulate strategies that emphasize visitor’s emotional resonance and enrich their spiritual experiences. For example, by creating interactive communication space, organizing cultural activities, and celebrating local festivals, visitors can be encouraged to deepen their understanding of local history and shape their unique memory of the place. Such approaches could trigger place attachment, thereby indirectly accelerating the actualization of the restorative potential in historical districts.

Furthermore, our findings provide compelling evidence that different visitor types hold disparate perceptions and emotional evaluations of historical districts. This insight can be leveraged effectively when crafting comprehensive strategies for the development, promotion, and management of historical districts. On the one hand, the development of historical districts should transition from a focus solely on esthetic preservation toward a holistic incorporation of everyday life, prioritizing local culture and fostering resident identification. On the other hand, by integrating cultural experiences into the business landscape and establishing leisurely cultural symbols within the district, which can increase perceptions and satisfaction for transient tourists.

### 5.3. Limitation and suggestions for further research

As in every study, this study is subject to several limitations. First, we employed four restorative environment characteristics (fascination, being away, scope, and coherence) as the second order manifest variable to assess the overall level of perceived restoration. Future studies can compare these factors as latent variables to facilitate a more detailed understanding of elements that support perceived recovery. Second, we acknowledge that human perception of physical settings is not solely visual; it is also influenced by the sounds they hear, and even more complicated environment they experienced. Thus, we propose further discussion to expand the possible contribution of soundscape or audio-visual interaction on perceived restoration in historical and cultural sites. Third, our investigation was conducted over a short period, however, it’s crucial to note that the conditions of the physical environment change long-term over time, much like how the landscape of a historic district might alter with the seasons. Therefore, future research can further apply the conceptual model in contrasting physical environments, which would enhance the multisensory exploration in restoration research. Forth, in current study, we did not specifically examine the potential impacts of demographics as control variables on the overall conceptual model, as it was not the primary research objectives. In future studies, it would be valuable to group factors such as gender, age, and education for comparative analysis to assess their influence regarding the relationship between landscape perception, emotional attachment, and restorative experiences. Furthermore, our research areas typically belong to residential historical districts. Thus, future studies could explore other types of historical districts, such as commercial and industrial historical districts, to further examine the generalizability of our proposed theoretical model.

## Data availability statement

The original contributions presented in the study are included in the article/supplementary material, further inquiries can be directed to the corresponding author.

## Ethics statement

Ethical review and approval were not required for the study on human participants in accordance with the local legislation and institutional requirements. Written informed consent to participate in this study was provided by the [patient/participants’ OR patient/participants legal guardian/next of kin].

## Author contributions

JiL and HW: conceptualization. JiL and TD: methodology. JiL, JuL, and JT: software. JiL and TD: formal analysis. JiL, TD, and JT: investigation. JiL and JuL: writing—original draft preparation. HW: writing—review and editing. All authors have read and agreed to the published version of this manuscript.

## Conflict of interest

The authors declare that the research was conducted in the absence of any commercial or financial relationships that could be construed as a potential conflict of interest.

## Publisher’s note

All claims expressed in this article are solely those of the authors and do not necessarily represent those of their affiliated organizations, or those of the publisher, the editors and the reviewers. Any product that may be evaluated in this article, or claim that may be made by its manufacturer, is not guaranteed or endorsed by the publisher.
