# Supplementary material for: Exploring perceived restoration, landscape perception, and place attachment in historical districts: insights from diverse visitors
Source: Front Psychol. 2023 Aug 2;14:1156207. doi: 10.3389/fpsyg.2023.1156207 (PMC10433909; doi:10.3389/fpsyg.2023.1156207)
Supplement: Supplementary file 1 [file Data_Sheet_1.docx]

Supplementary Material

Exploring perceived restoration, landscape perception, and place attachment in historical districts: insights from diverse visitors

Jiaying Li, Junjie Luo, Tangmin Deng, Jingwen Tian, and Hongcheng Wang *

*** Correspondence:** Hongcheng Wang: hongcheng_wang_tju@163.com

**Table A1**. MICOM: Compositional invariance.

|  | LR-SR | | LR-TT | | SR-TT | | Compositional invariance? |
| --- | --- | --- | --- | --- | --- | --- | --- |
| Composite | Original correlation | 95% CI | Original correlation | 95% CI | Original correlation | 95% CI |  |
| Perceived restoration | 1 | [1;1] | 1 | 1，1 | 0.977 | [1,1] | Yes/ Yes/ Yes |
| Landscape perception | 0.999 | [0.998;0.999] | 0.997 | [0.996;0.998] | 0.998 | [0.995;0.998] | Yes/ Yes/ Yes |
| Place identity | 1 | [0.999;1] | 1 | [0.999;1] | 1 | [1;1] | Yes/ Yes/ Yes |
| Place dependence | 1 | [0.999;1] | 1 | [0.999;1] | 0.999 | [0.998;1] | Yes/ Yes/ Yes |

Note: CI=confidence interval, LR = long-time residents, SR = short-time residents, TT = transient tourists.

**Table A2**. MICOM: Equal means

|  | LR-SR | | LR-TT | | SR-TT | | Equal means? |
| --- | --- | --- | --- | --- | --- | --- | --- |
| Composite | Original Differences | 95% CI | Original Differences | 95% CI | Original Differences | 95% CI |  |
| Perceived restoration | 0.172 | [-0.157;0.162] | 0.022 | [-0.200;0.196] | -0.157 | [-0.174;0.181] | **No**/Yes/Yes |
| Landscape perception | -0.058 | [-0.162;0.162] | -0.229 | [-0.200;0.193] | -0.193 | [-0.176;0.178] | Yes/**No**/**No** |
| Place identity | 0.207 | [-0.160;0.163] | 0.113 | [-0.200;0.201] | -0.096 | [-0.181;0.180] | **No**/Yes/Yes |
| Place dependence | 0.161 | [-0.159;0.163] | 0.071 | [-0.194;0.197] | -0.097 | [-0.176;0.175] | Yes/Yes/Yes |

Note: CI=confidence interval, LR = long-time residents, SR = short-time residents, TT = transient tourists.

**Table A3**. MICOM: Equal variances

|  | LR-SR | | LR-TT | | SR-TT | | Equal variances? |
| --- | --- | --- | --- | --- | --- | --- | --- |
| Composite | Original Differences | 95% CI | Original Differences | 95% CI | Original Differences | 95% CI |  |
| Perceived restoration | 0.139 | [-0.269;0.264] | 0.222 | [-0.354;0.378] | 0.081 | [-0.271;0.297] | Yes/Yes/Yes |
| Landscape perception | 0.376 | [-0.264;0.251] | 0.446 | [-0.318;0.338] | 0.073 | [-0.269;0.306] | **No**/**No/**Yes |
| Place identity | -0.240 | [-0.233;0.225] | -0.146 | [-0.270;0.290] | 0.091 | [-0.234;0.263] | **No**/Yes/Yes |
| Place dependence | -0.133 | [-0.260;0.223] | 0.254 | [-0.281;0.302] | 0.386 | [-0.249;0.274] | Yes/Yes/**No** |

Note: CI=confidence interval, LR = long-time residents, SR = short-time residents, TT = transient tourists.
